# 

**Published:** 1894-02

**Authors:** 


### CONTENTS.

PROCEEDINGS.

World's Columbian Dental Congress—1893.................................. 53

COMMUNICATIONS.

Sulphuric Acid for Opening Root Canals.................................. 73
The Relation of Dental Caries to Certain Forms of Indigestion...... 78
The "Devitalization of Pulps."—A Confession.............................. 82
"Vis Medicatrix Naturæ"............................................................ 86
A New Practical Method of Fusing Platinum.............................. 90
The Teeth of Children.................................................................. 92

EDITORIAL.

A New Degree.............................................................................. 100
Bibliographical............................................................................ 101
The American Medical Association.............................................. 102
Editorial Retirement.................................................................... 102
In Memory of Dr. Samuel Wardle................................................. 103

